# Racial Disparities in Cigarette Smoking Behaviors and Differences Stratified by Metropolitan Area of Residence

**DOI:** 10.3390/ijerph19052910

**Published:** 2022-03-02

**Authors:** Rony F. Arauz, Margaret Mayer, Carolyn Reyes-Guzman, Bríd M. Ryan

**Affiliations:** 1 Laboratory of Human Carcinogenesis, Center for Cancer Research, National Cancer Institute, Bethesda, MD 20892, USA; rony.arauz@gmail.com; 2 Tobacco Control Research Branch, Behavioral Research Program, Division of Cancer Control and Population Sciences, National Cancer Institute, Bethesda, MD 20892, USA; margaret.mayer@nih.gov (M.M.); carolyn.reyes-guzman@nih.gov (C.R.-G.)

**Keywords:** tobacco, cancer disparities, rural

## Abstract

Background: Black cigarette smokers experience a disproportionate burden of non-small cell lung cancer (NSCLC) compared to other racial and ethnic groups, despite starting to smoke later in life, smoking less frequently, and smoking fewer cigarettes per day compared with White smokers. Research has shown that these disparities in NSCLC are wider in rural areas. Objective: To examine differences in smoking behaviors between Black and White individuals living in non-metropolitan areas and metropolitan areas. Methods: Using harmonized data from the Tobacco Use Supplement to the Current Population Survey (TUS-CPS) years 2010–2011, 2014–2015, and 2018–2019, we compared smoking behaviors between Black and White current and former smokers by metropolitan status (i.e., whether an individual lives in a densely populated area or not) and by both metropolitan status and sex. Results: Smoking prevalence was higher among White participants living in non-metropolitan versus Black participants. Further, in non-metropolitan areas, Black individuals reported smoking fewer cigarettes per day, fewer years of smoking, and a later age of initiation compared to White individuals. Additionally, Black individuals, especially men, were more likely than White individuals to be current non-daily smokers. Conclusions: Our findings show that Black individuals living in non-metropolitan areas do not, in aggregate, have more cigarette smoking exposure relative to White individuals. Additional research is needed to further understand smoking-related exposures and other factors that may contribute to lung cancer disparities, especially in non-metropolitan areas.

## 1. Introduction

Lung cancer is the leading cause of cancer death in the United States (US) [1]. Incidence and mortality of lung cancer are higher among Black men compared with White men [2], and Black individuals in the US experience a disproportionate burden of lung cancer [3]. Although the mechanisms underlying this disparity are not fully understood, it is likely that they are driven by differences in cigarette smoking behaviors, as cigarette smoking is the leading cause of lung cancer [4]. For example, Black smokers are more likely to be light or non-daily smokers [5,6], start smoking at an older age [7], and consume fewer cigarettes per day (CPD) compared to White smokers [8]. Despite this, Black individuals experience lower rates of smoking cessation [9,10]. In addition, Black smokers have historically been more likely to use mentholated cigarettes, with 70–85% reporting usual use of menthol cigarettes, compared to approximately 20–30% of White smokers [11]. By changing inhalation patterns [12,13], increasing nicotine dependence [14], and inhibiting smoking cessation [15,16,17], menthol-flavored cigarettes can affect smoking behavior. Yet, studies have not shown an elevated risk of lung cancer among menthol cigarette smokers compared to non-menthol cigarette smokers [18,19], although small sample sizes of incident lung cancer cases among menthol cigarette smokers may have attenuated effect sizes in these studies. Black smokers may also have greater internal exposure to tobacco-related carcinogens per cigarette smoked, which could explain the higher incidence of lung cancer in this population [20]. However, whether and to what extent lung cancer disparities are driven by differences in internal exposure to carcinogens is unknown.

Recently, we reported that the degree of disparity between Black and White individuals in incidence of non-small cell lung cancer (NSCLC), which represents approximately 84% of all lung cancers [21], increased with increasing rurality of residence, with Black individuals having higher incidences rates [22]. Specifically, adenocarcinoma and squamous cell carcinoma incidence rates were more disparate in rural counties compared with metropolitan counties, and the differences in the degree of disparity were wider among men than among women. A growing body of research has explored urban/rural disparities in tobacco use. Studies have found that the overall prevalence of cigarette smoking, including both past 30-day and daily smoking, is significantly higher in rural areas compared to urban areas [23,24,25,26]. Furthermore, the prevalence of cigarette use is declining more slowly among rural populations than among urban populations, even after controlling for numerous smoking-related risk factors such as psychosocial and demographic characteristics [23]. However, no research has compared smoking behaviors between Black and White individuals across varying levels of rurality. If there are distinct tobacco use patterns among Black individuals compared to White individuals living in rural areas, this could help explain our recent findings. In this study, we therefore compared a comprehensive set of smoking behaviors and exposures between Black and White persons by metropolitan status (i.e., whether an individual lives in a densely populated area or not) and sex. Given that racial disparities in lung cancer incidence are widest in rural counties (with rural Black populations being most vulnerable), particularly among men [22], and given that tobacco use is the leading cause of lung cancer [27], we hypothesized that lifelong (cumulative) cigarette smoking exposure (e.g., years of smoking, CPD, and nicotine dependence) is greater among Black individuals, particularly men, living in non-metropolitan areas compared with White individuals living in non-metropolitan areas.

## 2. Methods

### 2.1. Data Source

Administered by the US Census Bureau and sponsored by the National Cancer Institute (NCI) and the Food and Drug Administration Center for Tobacco Products, the Tobacco Use Supplement to the Current Population Survey (TUS-CPS) is the largest cross-sectional survey of tobacco use among US adults (aged 18 years and older). During each wave of data collection, TUS-CPS uses multistage probability sampling to survey about 200,000 adults, producing a representative sample of the US civilian noninstitutionalized population. Since 1992, TUS-CPS has been conducted periodically to assess many tobacco-related topics, including tobacco use behaviors and history of tobacco product use, among others. Additional information about the TUS-CPS survey is available on the https://cancercontrol.cancer.gov/sites/default/files/2021-05/TUS-CPSFactsheet-508-05.13.21.pdf.

We used cigarette smoking behavior and sociodemographic variables from the harmonized TUS-CPS dataset [28], which offers larger sample sizes and more consistent variables over time. However, because the survey has changed over time, we pooled data from the three most recent survey waves (2010–2011, 2014–2015, 2018–2019), across which our variables of interest were consistent [28].

### 2.2. Measures

All participants were classified according to their current smoking status. Never smokers were defined as individuals who had smoked less than 100 cigarettes in their lifetime. Ever smokers were defined as individuals who had smoked greater than or equal to 100 cigarettes in their lifetime. Based on their response to the question, “do you now smoke every day, some days, or not at all?” ever smokers were further classified into current daily, current non-daily, and former smokers, respectively.

Smoking-related variables analyzed among current daily smokers were average number of cigarettes smoked per day (CPD, continuous); years of smoking every day (“smoking duration,” continuous, years); time to first cigarette after waking (TTFC, within 30 min/after 30 min/varies); regular use of menthol cigarettes (yes/no); age at smoking initiation (continuous, years); and having made a quit attempt in the past 12 months (yes/no). Smoking-related variables analyzed among non-daily smokers were number of days smoked cigarettes in the past 30 days (continuous); TTFC (within/after 30 min); regular use of menthol cigarettes (yes/no); and age at smoking initiation (continuous, years). Smoking-related variables analyzed among former smokers were TTFC during the year prior to quitting smoking (nicotine dependence prior to quitting, categorized as within/after 30 min); age at smoking initiation (continuous); and years since quitting completely (continuous).

Respondents were categorized as Black or White if they selected only Black or only White, respectively, from the list of races included in the demographic portion of CPS. Individuals reporting more than one race were excluded from analyses. Racial groups were determined regardless of Hispanic ethnicity or country of birth. As such, Black individuals include Hispanic and non-Hispanic persons, as well as those born in the US and elsewhere. Similarly, White individuals include Hispanic and non-Hispanic persons and White persons born inside and outside the US. Based on their county of residence, respondents were classified as living in either a metropolitan or non-metropolitan area, according to the US Census Bureau definitions [29]. Counties are classified as metropolitan based on their proximity to a densely populated urban core. Some individuals (n = 4493, 0.8%) were not classified as residing in either a metropolitan or non-metropolitan area because there were not enough individuals in their county to guarantee protection from deidentification. This group is not included in these analyses.

Sociodemographic characteristics included in these analyses were sex (male/female), age (18–24/25–34/35–54/≥55), educational attainment (0–8 years/9–12 years, no high school degree/high school degree/some college, including Associate’s/college graduate/postgraduate degree), yearly income (<USD 15,000/USD 15,000–24,999/USD 25,000–34,999/USD 35,000–49,999/USD 50,000–74,999/≥USD 75,000), region of the US (Northeast/Midwest/South/West), and survey wave (year).

### 2.3. Statistical Analysis

Accounting for complex survey design, we compared sociodemographic and survey characteristics (sex, age group, education level, income level, geographic region, and survey wave) between Black and White individuals by metropolitan status. For categorical smoking-related variables, we generated frequency estimates and weighted proportions and tested for differences by race with Rao-Scott Chi-square tests using SAS PROC SURVEYFREQ. For continuous smoking-related variables, we estimated weighted means and tested for differences by race with F-tests using SAS PROC SURVEYREG. To explore differences by sex, we repeated these analyses separately among men and women.

Analyses included TUS-CPS self-response survey weights, which are specific to each survey wave and account for complex sampling design and self-response. Prior to analysis, we adjusted the weights to reflect the combination of survey waves being pooled, such that the weighted population total represented the average US population for that combination of years. To estimate robust variances, we included TUS-CPS replicate weights in our analyses and adjusted them accordingly. All tests were two-sided, and a *p*-value of <0.05 was considered statistically significant. We performed all data analyses using SAS version 9.4 (SAS Institute, Inc., Cary, NC, USA).

## 3. Results

### 3.1. Description of the Study Population

Our analytic sample of 435,431 individuals included 339,649 residents of metropolitan areas (14.4% Black; 85.6% White) and 95,782 residents of non-metropolitan areas (8.4% Black; 91.6% White). In non-metropolitan areas, more than half of Black residents (55.3%) and White residents (52.6%) were women (Table 1). Additionally, in non-metropolitan areas, compared with Black individuals, White individuals tended to be older (Black individuals age 55 and older, 34.9%; White individuals age 55 and older, 42.9%). Further, compared with White residents of non-metropolitan areas, Black residents tended to report lower educational attainment and a lower annual income. The vast majority (90.6%) of Black residents of non-metropolitan areas lived in the southern region of the US, as did a majority (52.9%) of Black residents of metropolitan areas. In contrast, White residents of both non-metropolitan and metropolitan regions were more evenly distributed across the four US regions.

### 3.2. Differences in Smoking Behaviors by Race and Metropolitan Status

Black residents of non-metropolitan areas were less likely than White residents to be ever smokers (28.3% vs. 40.4%, respectively; Table 2) and less likely to be current daily smokers (12.8% vs. 15.7%). In contrast, Black residents were more likely to be current non-daily smokers compared with White residents (4.7% vs. 3.0%). Among daily smokers living in non-metropolitan areas, Black persons, compared to White persons, reported smoking fewer cigarettes per day (12.4 CPD vs. 16.6 CPD, *p* < 0.0001), fewer years of smoking (23.0 years vs. 25.4 years, *p* = 0.002), and a later age of smoking initiation (18.6 years vs. 17.2 years, *p* < 0.0001). Black daily smokers were less likely than White daily smokers to report smoking their first cigarette within 30 min of waking (56.4% vs. 64.2%, *p* < 0.0001), and there was no significant difference in the proportion reporting that they had made a past-year quit attempt (36.3% vs. 33.1%, *p* = 0.1204). However, most Black daily smokers living in non-metropolitan areas reported currently smoking mentholated cigarettes (82.1% vs. 35.6% for White smokers, *p* < 0.0001).

Among non-daily smokers living in non-metropolitan areas, there was no significant difference in the average number of days smoked out of the past 30 days (Black individuals 14.1 days vs. White individuals 14.4 days, *p* = 0.6448) or in the proportion reporting that they smoked their first cigarette within 30 min of waking (Black individuals 16.2% vs. White individuals 13.6%, *p* = 0.5296). Black non-daily smokers, compared to White non-daily smokers, reported a later age of smoking initiation (20.9 years vs. 18.7 years, *p* < 0.0001). However, Black non-daily smokers living in non-metropolitan areas were more likely than White non-daily smokers to report smoking mentholated cigarettes (87.8% vs. 35.7%, *p* < 0.0001).

Among former smokers living in non-metropolitan areas, there was no significant difference in the proportion reporting that they smoked their first cigarette within 30 min of waking during the year prior to quitting (Black individuals 50.6% vs. White individuals 50.7%, *p* = 0.6787). Further, Black former smokers, compared to White former smokers, reported an older age of cigarette initiation (18.6 years vs. 17.7 years, *p* < 0.0001) but fewer years since quitting smoking (15.3 years vs. 18.9 years, *p* < 0.0001). We observed similar patterns among residents of metropolitan areas.

### 3.3. Differences in Smoking Patterns by Metropolitan Status and Sex

#### 3.3.1. Men

Among residents of non-metropolitan areas, Black men were less likely than White men to be ever smokers (36.6% vs. 45.7%, respectively; Table 3), but they were more likely to be current smokers (22.6% vs. 19.7%) and current non-daily smokers (6.1% vs. 3.3%). Among daily smokers living in non-metropolitan areas, Black men, compared to White men, reported smoking fewer cigarettes per day (12.9 CPD vs. 18.1 CPD, *p* < 0.0001), fewer years of smoking (24.1 years vs. 26.4 years, *p* = 0.0151), and a later age of smoking initiation (18.3 years vs. 16.8 years, *p* < 0.0001). Further, Black men were less likely to report smoking their first cigarette within 30 min of waking (55.5% vs. 66.2% among White men, *p* < 0.0001) and more likely to report a past-year quit attempt (36.9% vs. 30.9% among White men, *p* = 0.0439). However, they were also more likely to report using mentholated cigarettes (83.0% vs. 30.5% among White men, *p* < 0.0001).

Among non-daily smokers living in non-metropolitan areas, there was no significant difference in the average number of days smoked out of the past 30 days (Black men 13.8 days vs. White men 14.6 days, *p* = 0.2778), the proportion reporting that they smoked their first cigarette within 30 min of waking (Black men 13.5% vs. White men 14.5%, *p* = 0.9445), or the average age of smoking initiation (Black men 20.4 years vs. White men 18.4 years, *p* = 0.0533). However, Black non-daily smokers living in non-metropolitan areas were more likely than White non-daily smokers to report smoking mentholated cigarettes (87.0% vs. 30.3%, *p* < 0.0001). We observed similar patterns among men living in metropolitan areas.

#### 3.3.2. Women

Among residents of non-metropolitan areas, Black women were less likely than white women to be ever smokers (21.7% vs. 35.6%, respectively; Table 4), current smokers (13.4% vs. 17.8%), and current daily smokers (9.8% vs. 15.1%). Among daily smokers living in non-metropolitan areas, Black women, compared to White women, reported smoking fewer cigarettes per day (11.8 CPD vs. 15.1 CPD, *p* < 0.0001), fewer years of smoking (21.5 years vs. 24.4 years, *p* = 0.0060), and a later age of smoking initiation (19.1 years vs. 17.6 years, *p* < 0.0001). Black women were less likely than White women to report smoking their first cigarette within 30 min of waking (57.6% vs. 62.3%, *p* < 0.0001), and there was no significant difference in the proportion of daily smokers who reported making a past-year quit attempt (Black women 35.4% vs. White women 35.2%, *p* = 0.9439). However, as with men, Black women living in non-metropolitan areas were more likely to report using mentholated cigarettes (80.9% vs. 40.7% among White women, *p* < 0.0001).

In contrast to what was observed among men, among women who lived in non-metropolitan areas, Black non-daily smokers were more likely than White non-daily smokers to report smoking their first cigarette of the day within 30 min of waking (20.1% vs. 12.7%, *p* = 0.0436), and Black non-daily smokers reported a later age of smoking initiation (21.4 years vs. 18.9 years, *p* = 0.0005). Consistent with what was observed among men, among women who lived in non-metropolitan areas, there was no significant difference between Black and White non-daily smokers in the number of days smoked out of the past 30 days (Black women 14.6 days vs. White women 14.2 days, *p* = 0.6640), and Black non-daily smokers were more likely to report using mentholated cigarettes (88.8% vs. 41.6% among White women, *p* < 0.0001). We observed similar patterns among women living in metropolitan areas

## 4. Discussion

The overarching aim of the present study was to examine differences in smoking behaviors between Black and White individuals by metropolitan area of residence. In aggregate, our results suggest that Black individuals living in non-metropolitan areas do not have increased cigarette smoking exposure compared with White individuals. However, we observed that certain behaviors were more prevalent among Black individuals. Black men were more likely than White men to be current smokers, however the difference was predominantly driven by a higher prevalence of non-daily smoking among Black individuals. Further, across subgroups of race, metropolitan status, sex, and frequency of smoking, Black individuals were more likely to report that they usually smoked menthol cigarettes compared with Whites. In some groups the prevalence of menthol cigarette use reached as high as 88.8%. Historically, the tobacco industry has employed many tactics to promote menthol cigarette use among Black individuals, including, among other approaches, the use of culturally tailored messages and images [30] in marketing and targeted advertising of menthol cigarettes in predominantly Black neighborhoods [31,32]. As previously discussed, use of menthol cigarettes is associated with altered inhalation patterns [12,13], greater nicotine dependence [14], and less smoking cessation [15,16,17]. Further, menthol has been shown to slow nicotine metabolism and clearance of certain carcinogens. However, cohort studies have not yet indicated a higher risk of lung cancer associated with smoking menthol cigarettes [18,19]. Further study with larger, more racially and ethnically diverse cohorts is likely needed.

Interestingly, across both metropolitan and non-metropolitan areas, we observed that White daily smokers were significantly more likely to report high-to-moderate nicotine dependence and smoked significantly more CPD on average than Black daily smokers. This is contrary to some evidence that nicotine dependence is higher among Black smokers compared with White smokers [33,34,35]. However, it is consistent with other research has found that White smokers tend to smoke more CPD than Black smokers [7]. Additionally, White smokers have faster rates of metabolizing nicotine when compared to Black smokers [33], thereby potentially resulting in higher nicotine addiction scores. Taken together, these observations add to the body of evidence that Black and White individuals have markedly different smoking behaviors, flavor preferences, such as menthol, and nicotine pharmacokinetics [3,20]. We recognize that, depending on the outcome variable, the groups used in our analyses treat race as a social and a biological construct, despite the controversies that such terminology has generated [36]. Still, more research in this area is needed to understand the intrapersonal associations between social influences (e.g., menthol cigarette marketing) and biological predispositions (e.g., nicotine metabolism), given that race can be a marker for both.

We also found that Black smokers in both metropolitan and non-metropolitan areas were more likely than White smokers to report making a quit attempt, which is consistent with prior research [37,38,39]. Research has found that Black individuals are less likely to receive smoking cessation advice from a healthcare provider [40,41], and are less likely to use prescription smoking cessation medications compared with white smokers [9,42]. Given the higher incidence of lung cancer among Black smokers, it is critical that healthcare providers counsel all patients, and particularly Black patients, on the health benefits of quitting all tobacco products, not only cigarettes. Clinicians should assess smoking status, advise smokers to quit, and assist their patients throughout the quitting process by encouraging the use of evidence-based cessation methods (both pharmacological and behavioral) as needed. Moreover, upstream factors such as provider mistrust [43] and quality of patient-physician communication [44], among others, must be addressed in order to make sustainable change. Yet, it is also critical for interventions to take into account issues of access in rural areas, including shortages of health professionals and longer distances to receive care, which may affect the frequency and nature of encounters with health care providers [45]. Programs such as NCI’s Smokefree.gov [46], which offers web- and text message-based assistance, may be helpful in these settings, as may policy interventions that address these barriers.

An important strength of this study is that it uses a robust and nationally representative sample of Black and White individuals. In addition, we examined pooled data from many years, included a range of detailed smoking behavior variables, and had a large sample size that allowed us to systematically compare many subgroups. There are, however, several limitations to consider. First, it was not possible to evaluate the temporal relationship between metropolitan status and smoking behaviors using a cross-sectional study design. However, the majority of our results were consistent with previous cross-sectional studies of tobacco use and smoking behaviors among Black individuals compared with White individuals in the US. Second, the TUS-CPS data are self-reported, so some data on smoking behavior rely on the respondents’ ability to recall past events, which may have rendered our study prone to recall bias. Third, while our sample size was large overall, the sample sizes of some subgroups of non-metropolitan residents were small which may have prevented us from detecting some differences. Further, small sample size for certain subgroups did not allow us to examine use of cigarettes together with other forms of tobacco, (e.g., cigars). Given that Black smokers are more likely than White smokers to co-use other tobacco products with cigarettes [47], it is possible that co-use behaviors could contribute to disparities in lung and other cancers. This issue is not addressed by our research and merits further examination. Fourth, the data used for these analyses were collected between 2010 and 2019. Some research has found that racial disparities in lung cancer incidence have been decreasing in younger cohorts [48]. Given the long latency period between smoking and lung cancer, analysis of historic tobacco use data may have identified different patterns from those presented here. Fifth, the race categories used for these analyses were broad and did not account for Hispanic ethnicity or country of birth; this, too, may have prevented us from detecting some differences. Lastly, metropolitan status does not necessarily correspond to urbanicity. Thus, it is possible that some trends by race and metropolitan status could have been masked.

In summary, our results indicate that for the time period of 2010–2019, Black smokers living in non-metropolitan areas did not, in aggregate, have more cigarette smoking exposure relative to White smokers. Consequently, cigarette use, as measured in this study, does not entirely explain the wider disparity in lung cancer incidence between Black and White individuals in non-metropolitan areas, compared with metropolitan areas. It is possible that other risk factors for lung cancer, such as secondhand smoke, radon, particle pollution [49,50,51], or genetics, may be driving this phenomenon, however additional work using longitudinal designs and exploring other tobacco use behaviors will be needed to further ascertain the smoking-related or other exposures that contribute to lung cancer disparities, especially in rural areas. In the interim, it remains critical that health care providers, public health practitioners, and policymakers employ evidence-based tobacco prevention and control strategies and that they tailor these interventions and policies to the populations that are disproportionately burdened by tobacco use.

## Figures and Tables

**Table 1:** Sociodemographic characteristics of participants by metropolitan and non-metropolitan residence (n = 435,431) ^1^.

Characteristics	Metropolitan (n = 339,649)			Non-Metropolitan (n = 95,782)		
	White (n = 298,303)	Black (n = 41,346)		White (n = 89,934)	Black (n = 5848)	
	Weighted % (95% CI) (n)	Weighted % (95% CI) (n)	*p*-Value	Weighted % (95% CI) (n)	Weighted % (95% CI) (n)	*p*-Value
**Gender**			<0.0001			<0.0001
Male	49.0 (48.8, 48.2) (135,722)	45.1 (44.6, 45.6) (16,457)		47.4 (47.1, 47.8) (40,065)	44.7 (43.4, 46.0) (2231)	
Female	51.0 (50.8, 51.2) (162,581)	54.9 (54.4, 55.4) (24,889)		52.6 (52.2, 52.9) (49,869)	55.3 (54.0, 56.6) (3617)	
**Age (years)**			<0.0001			<0.0001
18–24	11.8 (11.7, 11.9) (19,271)	15.2 (14.8, 15.5) (3563)		11.3 (10.9, 11.7) (5381)	15.7 (14.5, 17.0) (495)	
25–34	17.5 (17.3, 17.6) (48,941)	20.5 (20.1, 20.9) (7372)		14.2 (13.9, 14.6) (11,827)	16.1 (14.8, 17.5) (684)	
35–54	34.4 (34.3, 34.6) (104,009)	35.8 (35.4, 36.3) (15,358)		31.6 (31.1, 32.0) (28,294)	33.3 (32.0, 34.6) (1983)	
≥55	36.3 (36.1, 36.5) (126,082)	28.5 (28.1, 28.9) (15,053)		42.9 (42.3, 43.5) (44,432)	34.9 (33.3, 36.5) (2506)	
**Education**			<0.0001			<0.0001
0–8 years	3.7 (3.6, 3.8) (9719)	2.5 (2.3, 2.7) (1213)		4.0 (3.7, 4.3) (3252)	5.2 (4.3, 6.1) (350)	
9–12 years, no high school degree	6.7 (6.6, 6.8) (17,437)	10.6 (10.2, 11.0) (4464)		8.8 (8.5, 9.2) (6870)	17.4 (15.9, 19.0) (1049)	
High school degree	26.2 (26.0, 26.5) (77,725)	31.3 (30.7, 31.9) (12,963)		37.1 (36.4, 37.7) (31,910)	40.6 (38.9,42.3) (2282)	
Some college, incl. associates degrees	28.6 (28.3, 28.8) (84,337)	33.1 (32.6, 33.7) (13,093)		30.0 (29.5, 30.6) (27,174)	27.1 (25.5, 28.7) (1554)	
College graduate	22.4 (22.3, 22.6) (68,524)	14.3 (13.9, 14.8) (5936)		13. 4 (12.9,13.9) (13,677)	6.6 (5.8, 7.5) (400)	
Postgraduate	12.4 (12.2, 12.6) (40,561)	8.2 (7.8, 8.5) (3677)		6.7 (6.4,7.0) (7051)	3.1 (2.7, 3.7) (213)	
**Income**			<0.0001			<0.0001
<USD 15,000	9.7 (9.5, 9.9) (24,167)	22.5 (21.9, 23.2) (8192)		14.6 (14.0,15.3) (10,509)	37.7 (35.7, 39.7) (1926)	
USD 15,000–24,999	8.9 (8.7, 9.1) (22,881)	12.9 (12.4, 13.3) (4581)		12.7 (12.3, 13.2) (9634)	19.5 (18.0, 21.0) (958)	
USD 25,000–34,999	10.5 (10.3, 10.7) (26,846)	13.5 (13.0, 14.0) (4766)		14.0 (13.5, 14.4) (10,320)	14.8 (13.5, 16.2) (742)	
USD 35,000–49,999	13.5 (13.3, 13.7) (34,468)	14.8 (14.3, 15.4) (5047)		15.7 (15.3, 16.2) (11,984)	11.7 (10.4, 13.2) (564)	
USD 50,000–74,999	18.9 (18.6, 19.1) (49,182)	15.9 (15.3, 16.5) (5356)		19.6 (19.0, 20.1) (15,242)	10.3 (8.9, 11.8) (514)	
≥USD 75,000	38.5 (38.2, 38.8) (97,371)	20.4 (19.8, 21.1) (6616)		23.4 (22.7, 24.2) (18,427)	6.0 (5.0, 7.2) (268)	
**Region**			<0.0001			<0.0001
Northeast	19.3 (19.0, 19.6) (58,085)	19.1 (18.8, 19.5) (6230)		12.2 (10.8, 13.6) (15,696)	2.0 (1.4, 2.9) (100)	
Midwest	20.6 (20.2, 21.0) (64,657)	17.9 (17.5,18.2) (6750)		36.3 (34.3, 38.4) (29,194)	5.9 (4.8, 7.3) (301)	
South	35.1 (34.6, 35.5) (98,648)	52.9 (52.5, 53.4) (24,471)		38.0 (35.8, 40.3) (26,320)	90.6 (88.9, 92.1) (5325)	
West	25.0 (24.6, 25.5) (76,913)	10.1 (9.8,10.4) (3895)		13.5 (11.8, 15.5) (18,724)	1.5 (1.1, 2.0) (122)	
**Survey year**			<0.0001			0.4972
2010–2011	32.0 (31.7, 32.3) (107,977)	30.5 (30.0, 30.9) (14,782)		35.8 (34.2, 37.4) (33,621)	34.0 (31.0, 37.2) (1912)	
2014–2015	33.3 (33.1, 33.5) (102,719)	33.5 (33.0, 33.9) (14,673)		33.5 (32.7, 34.2) (31,403)	34.6 (32.5, 36.7) (2192)	
2018–2019	34.7 (34.4, 35.0) (87,607)	36.0 (35.6, 36.5) (11,891)		30.7 (29.1, 32.4) (24,910)	31.4 (28.1, 34.9) (1744)	

^1^ Frequencies are unweighted; percentages and statistical tests are weighted. To compare sample characteristics between Black and White individuals living in metropolitan areas and between Black and White individuals living in non-metropolitan areas, Rao-Scott Chi-square tests and F-tests were used for categorical and continuous variables, respectively.

**Table 2:** Population distribution of smoking behaviors by metropolitan and non-metropolitan residence among all participants (n = 435,431) ^1^.

Smoking Behaviors	Metropolitan (n = 339,649)			Non-Metropolitan (n = 95,782)		
	White	Black		White	Black	
	Weighted % (95% CI) (n)		*p*-Value	Weighted % (95% CI) (n)		*p*-Value
Smoking status			<0.0001 ^2^			<0.0001 ^2^
Never	66.8 (66.6, 67.1) (191,025)	75.1 (74.6, 75.6) (29,748)		59.6 (58.9, 60.3) (52,423)	71.7 (69.8, 73.5) (4072)	
Ever	33.2 (32.9, 33.4) (105,758)	24.9 (24.4, 25.4) (11,340)		40.4 (39.7, 41.1) (37,091)	28.3 (26.5, 30.2) (1740)	
Former	20.2 (20.0, 20.3) (66,286)	11.0 (10.7, 11.3) (5230)		21.7 (21.2, 22.2) (21,309)	10.8 (9.9, 11.8) (722)	
Current	13.0 (12.8, 13.2) (39,472)	13.9 (13.5, 14.3) (6110)		18.7 (18.2, 19.2) (15,782)	17.5 (16.0, 19.0) (1018)	
Current daily	10.1 (10.0, 10.3) (31,190)	9.9 (9.6, 10.3) (4378)		15.7 (15.2, 16.2) (13,235)	12.8 (11.6, 14.0) (753)	
Current non-daily	2.9 (2.8, 2.9) (8282)	4.0 (3.8, 4.2) (1732)		3.0 (2.8, 3.2) (2547)	4.7 (3.9, 5.6) (265)	
Daily Smokers						
Average number of cigarettes per day (weighted mean ± SD, n)	15.0 ± 0.06 (30,515)	11.0 ± 0.12 (4216)	<0.0001	16.6 ± 0.12 (12,985)	12.4 ± 0.34 (734)	<0.0001
Smoking duration (years, mean ± SD, n)	24.9 ± 0.11 (29,774)	23.7 ± 0.27 (4064)	<0.0001	25.4 ± 0.18 (12,673)	23.0 ± 0.75 (706)	0.0020
Smoke first cigarette of the day within 30 min of waking			<0.0001			<0.0001
Yes	56.6 (55.8, 57.3) (17,550)	52.3 (50.6, 54.1) (2257)		64.2 (62.9, 65.5) (8304)	56.4 (52.2, 60.4) (423)	
No	41.8 (41.1, 42.5) (12,448)	44.9 (43.2, 46.6) (1871)		34.6 (33.4, 35.7) (4519)	39.2 (35.3, 43.2) (287)	
Varies	1.6 (1.5, 1.8) (476)	2.8 (2.3, 3.4) (132)		1.2 (1.0, 1.6) (158)	4.4 (3.0, 6.5) (32)	
Menthol cigarette use			<0.0001			<0.0001
Yes	39.1 (38.4, 39.8) (11,260)	84.5 (83.0, 85.9) (3605)		35.6 (34.4, 36.9) (4299)	82.1 (78.3, 85.4) (608)	
No	60.9 (60.2, 61.6) (19,071)	15.5 (14.1, 17.0) (658)		64.4 (63.1, 65.6) (8639)	17.9 (14.6, 21.7) (132)	
Age at smoking initiation (years, weighted mean ± SD, n)	17.5 ± 0.03 (30,431)	18.3 ± 1.00 (4179)	<0.0001	17.2 ± 0.06 (12,927)	18.6 ± 0.17 (720)	<0.0001
Made a quit attempt in the past 12 months			<0.0001			0.1204
Yes	35.4 (34.8, 36.0) (10,655)	39.9 (38.3, 41.6) (1632)		33.1 (32.0, 34.2) (4401)	36.3 (32.5, 40.2) (267)	
No	64.6 (64.0, 65.2) (19,583)	60.1 (58.4, 61.7) (2584)		66.9 (65.8, 68.0) (8476)	63.7 (59.8, 67.5) (466)	
Non-Daily Smokers						
Number of days smoked out of the past 30 days (mean ± SD, n)	13.4 ± 0.10 (7618)	14.2 ± 0.24 (1578)	0.0014	14.4 ± 0.23 (2363)	14.1 ± 0.50 (243)	0.6448
Smoke first cigarette of the day within 30 min of waking			<0.0001			0.5296
Yes	10.3 (9.5, 11.1) (884)	17.5 (15.3, 19.9) (288)		13.6 (12.1, 15.3) (351)	16.2 (10.8, 23.7) (42)	
No	86.1 (85.2, 87.1) (6932)	78.0 (75.5, 80.4) (1310)		82.9 (80.9, 84.7) (2053)	79.3 (71.4, 85.5) (200)	
Varies	3.6 (3.1, 4.1) (278)	4.5 (3.5, 5.7) (82)		3.5 (2.7, 4.6) (91)	4.5 (2.2, 8.7) (11)	
Menthol cigarette use			<0.0001			<0.0001
Yes	37.0 (35.7, 38.4) (2889)	80.6 (78.1, 82.9) (1391)		35.7 (33.1, 38.4) (821)	87.8 (81.2, 92.3) (225)	
No	63.0 (61.6, 64.3) (5185)	19.4 (17.1, 21.9) (292)		64.3 (61.6, 66.9) (1672)	12.2 (7.7, 18.8) (32)	
Age at smoking initiation (years, weighted mean ± SD, n)	18.9 ± 0.06 (7773)	20.0 ± 0.16 (1638)	<0.0001	18.7 ± 0.12 (2396)	20.9 ± 0.70 (251)	0.002
Former Smokers						
During year before made a quit attempt, smoke first cigarette of the day within 30 min of waking			0.0789			0.6787
Yes	41.8 (40.7, 43.0) (4039)	42.7 (39.0, 46.4) (387)		50.7 (48.6, 52.8) (1521)	50.6 (40.0, 61.2) (79)	
No	55.6 (54.4, 56.7) (5028)	53.3 (49.4, 57.1) (471)		47.0 (44.9, 49.1) (1375)	45.8 (35.0, 56.9) (55)	
Varies	2.6 (2.2, 3.0) (221)	4.0 (2.8, 5.8) (41)		2.3 (1.7, 3.1) (61)	3.6 (1.5, 8.1) (7)	
Age at smoking initiation (years, mean ± SD, n)	17.8 ± 0.02 (62,835)	18.6 ± 0.08 (4865)	<0.0001	17.7 ± 0.05 (20,248)	18.6 ± 0.20 (682)	<0.0001
Years since completely stopped smoking (mean ± SD, n)	18.7 ± 0.06 (65,395)	15.9 ± 0.23 (5100)	<0.0001	18.9 ± 0.61 (21,044)	15.3 ± 0.61 (715)	<0.0001

^1^ Frequencies are unweighted; percentages and statistical tests are weighted. To compare sample characteristics between Black and White individuals living in metropolitan areas and between Black and White individuals living in non-metropolitan areas, Rao-Scott Chi-square tests and F-tests were used for categorical and continuous variables, respectively. ^2^ Tests for differences in current smoking status (i.e., current/former/never).

**Table 3:** Population distribution of smoking behaviors by metropolitan and non-metropolitan residence among men (n = 194,475) ^1^.

Smoking Behaviors	Metropolitan (n = 152,179)			Non-Metropolitan (n = 42,296)		
	White	Black		White	Black	
	Weighted % (95% CI) (n)		*p*-Value	Weighted % (95% CI) (n)		*p*-Value
Smoking status			<0.0001 ^2^			<0.0001 ^2^
Never	62.7 (62.4, 63.0) (80,455)	70.2 (69.4, 70.9) (10,793)		54.3 (53.4, 55.1) (20,943)	63.4 (61.0, 65.8) (1309)	
Ever	37.3 (37.0, 37.6) (54,515)	29.8 (29.1, 30.6) (5552)		45.7 (44.9, 46.6) (18,909)	36.6 (34.2, 39.0) (902)	
Former	22.8 (22.6, 23.1) (34,666)	13.0 (12.61, 13.6) (2598)		26.0 (25.4, 26.7) (11,452)	14.0 (12.6, 15.5) (373)	
Current	14.5(14.2, 14.7) (19,849)	16.8 (16.1, 17.5) (2954)		19.7 (19.1, 20.3) (7457)	22.6 (20.5, 24.8) (529)	
Current daily	11.1 (10.9, 11.3) (15,555)	11.9 (11.3, 12.5) (2116)		16.4 (15.8, 17.0) (6196)	16.5 (14.7, 18.5) (390)	
Current non-daily	3.4 (3.3, 3.5) (4294)	4.9 (4.5, 5.3) (838)		3.3 (3.1, 3.6) (1261)	6.1 (4.76, 7.7) (139)	
Missing (n)	752	112		213	20	
**Daily Smokers**						
Average number of cigarettes per day (weighted mean ± SD, n)	16.1 ± 0.08 (15,193)	11.8 ± 0.17 (2046)	<0.0001	18.1 ± 0.17 (6068)	12.9 ± 0.46 (380)	<0.0001
Smoking duration (years, mean ± SD, n)	25.0 ± 0.15 (14,819)	24.0 ± 0.39 (1963)	0.0182	26.4 ± 0.26 (5924)	24.1 ± 1.00 (366)	0.0151
Smoke first cigarette of the day within 30 min of waking			<0.0001			<0.0001
Yes	57.1 (56.2, 58.1) (8903)	51.0 (48.5, 53.4) (1074)		66.2 (64.6, 67.7) (4005)	55.5 (49.8, 61.0) (216)	
No	41.2 (40.2, 42.1) (6024)	46.0 (43.5, 48.2) (915)		32.4 (30.9, 33.9) (1985)	40.1 (34.6, 45.8) (152)	
Varies	1.7 (1.4, 1.9) (243)	3.0 (2.4, 3.9) (73)		1.4 (1.1, 1.9) (83)	4.4 (2.7, 7.2) (17)	
Menthol cigarette use			<0.0001			<0.0001
Yes	34.6 (33.7, 35.4) (4849)	82.0 (79.8, 84.0) (1671)		30.5 (28.8, 32.1) (1718)	83.0 (77.9, 87.2) (312)	
No	65.4 (64.6, 66.3) (10,236)	18.0 (16.0, 20.2) (390)		69.5 (67.9, 71.2) (4321)	17.0 (12.8, 22.1) (71)	
Age at smoking initiation (years, weighted mean ± SD, n)	17.2 ± 0.05 (15,155)	17.9 ± 0.12 (2019)	<0.0001	16.8 ± 0.09 (6047)	18.3 ± 0.22 (372)	<0.0001
Made a quit attempt in the past 12 months			0.0007			0.0439
Yes	34.1 (33.3, 34.9) (5111)	38.6 (36.1, 41.0) (745)		30.9 (29.5, 32.4) (939)	36.9 (31.5, 42.8) (141)	
No	65.9 (65.0, 66.7) (9944)	61.4 (59.0, 63.9) (1292)		69.1 (67.6, 70.5) (4079)	63.1 (57.2, 68.5) (241)	
Non-Daily Smokers						
Number of days smoked out of the past 30 days (mean ± SD, n)	13.2 ± 0.14 (3947)	14.2 ± 0.36 (756)	0.009	14.6 ± 0.33 (1170)	13.8 ± 0.60 (124)	0.2778
Smoke first cigarette of the day within 30 min of waking			0.0012			0.9445
Yes	10.2 (9.2, 11.3) (457)	14.9 (12.1, 18.1) (116)		14.5 (12.3, 17.0) (185)	13.5 (8.5, 20.7) (20)	
No	85.9 (84.6, 87.2) (3585)	79.9 (76.3, 83.1) (648)		81.6 (78.8, 84.0) (998)	83.0 (74.7, 89.1) (109)	
Varies	3.9 (3.3, 4.7) (161)	5.2 (3.7, 7.2) (46)		3.9 (2.8, 5.6) (50)	3.5 (1.0, 11.7) (4)	
Menthol cigarette use			<0.0001			<0.0001
Yes	33.1 (31.3, 34.9) (1282)	77.8 (74.0, 81.2) (647)		30.3 (26.8, 34.0) (342)	87.0 (77.3, 93.0) (121)	
No	66.9 (65.1, 68.7) (2901)	22.2 (18.8, 26.0) (164)		69.7 (66.0, 73.2) (888)	13.0 (7.0, 22.7) (15)	
Age at smoking initiation (years, weighted mean ± SD, n)	18.7 ± 0.08 (4015)	19.4 ± 0.20 (790)	0.0004	18.4 ± 0.16 (1172)	20.4 ± 1.02 (133)	0.0533
Former Smokers						
During year before made a quit attempt, smoke first cigarette of the day within 30 min of waking			0.2073			0.9127
Yes	42.4 (40.8, 44.0) (2098)	40.7 (35.7, 45.9) (173)		53.0 (49.8, 56.2) (785)	52.2 (37.5, 66.5) (37)	
No	55.2 (53.6, 56.8) (2490)	55.3 (50.1, 60.4) (230)		44.3 (41.2, 47.5) (635)	45.9 (31.7, 60.8) (23)	
Varies	2.4 (2.0, 3.0) (116)	4.0 (2.3, 7.0) (17)		2.7 (1.8, 4.0) (37)	1.9 (0.4, 9.0) (2)	
Age at smoking initiation (years, mean ± SD, n)	17.5 ± 0.03 (32,913)	18.2 ± 0.11 (2412)	<0.0001	17.2 ± 0.05 (10,896)	18.0 ± 0.33 (355)	0.0120
Years since completely stopped smoking (mean ± SD, n)	18.9 ± 0.08 (34,195)	16.3 ± 0.33 (2536)	<0.0001	19.7 ± 0.21 (11,321)	15.2 ± 0.82 (370)	<0.0001

^1^ Frequencies are unweighted; percentages and statistical tests are weighted. To compare sample characteristics between Black and White individuals living in metropolitan areas and between Black and White individuals living in non-metropolitan areas, Rao-Scott Chi-square tests and F-tests were used for categorical and continuous variables, respectively. ^2^ Tests for differences in current smoking status (i.e., current/former/never).

**Table 4:** Population distribution of smoking behaviors by metropolitan and non-metropolitan residence among women (n = 240,956) ^1^.

Smoking Behaviors	Metropolitan (n = 187,470)			Non-Metropolitan (n = 53,486)		
	White	Black		White	Black	
	Weighted % (95% CI) (n)		*p*-Value	Weighted % (95% CI) (n)		*p*-Value
Smoking status			<0.0001 ^2^			<0.0001 ^2^
Never	70.8 (70.6, 71.1) (110,570)	79.1 (78.5, 79.7) (18,955)		64.4 (63.6, 65.1) (31,480)	78.3 (76.1, 80.4) (2763)	
Ever	29.2 (28.9, 29.4) (51,243)	20.9 (20.3, 21.5) (5788)		35.6 (34.9, 36.4) (18,182)	21.7 (19.6, 23.9) (838)	
Former	17.6 (17.4, 17.8) (31,620)	9.3 (9.0, 9.7) (2632)		17.8 (17.3, 18.4) (9857)	8.3 (7.3, 9.5) (349)	
Current	11.6 (11.4, 11.8) (19,623)	11.6 (11.1, 12.0) (3156)		17.8 (17.2, 20.0) (8325)	13.4 (11.8, 15.1) (489)	
Current daily	9.2 (9.1, 9.4) (15,635)	8.3 (7.9, 8.7) (2262)		15.1 (14.5, 15.7) (7039)	9.8 (8.6, 11.1) (363)	
Current non-daily	2.4 (2.3, 2.4) (3988)	3.3 (3.0, 3.5) (894)		2.7 (2.5, 2.9) (1286)	3. 6 (2.9, 4.5) (126)	
Missing (n)	768	146		207	16	
Daily Smokers						
Average number of cigarettes per day (weighted mean ± SD, n)	13.7 ± 0.08 (15,322)	10.1 ± 0.15 (2170)	<0.0001	15.1 ± 0.14 (6917)	11.8 ± 0.50 (354)	<0.0001
Smoking duration (years, mean ± SD, n)	24.8 ± 0.15 (14,955)	23.3 ± 0.37 (2101)	0.0002	24.4 ± 0.24 (6749)	21.5 ± 1.02 (340)	0.0060
Smoke first cigarette of the day within 30 min of waking			0.0202			<0.0001
Yes	55.9 (54.9, 56.9) (8647)	53.9 (51.4, 56.5) (1183)		62.3 (60.6, 63.9) (4299)	57.6 (50.6, 64.2) (207)	
No	42.5 (41.5, 43.5) (6424)	43.5 (41.1, 46.0) (956)		36.6 (35.0, 38.3) (2534)	38.0 (31.7, 44.7) (135)	
Varies	1.6 (1.4, 1.9) (233)	2.6 (1.9, 3.4) (59)		1.1 (0.8, 1.4) (75)	4.4 (2.6, 7.5) (15)	
Menthol cigarette use			<0.0001			<0.0001
Yes	44.3 (43.4, 45.2) (6411)	87.6 (85.8, 89.1) (1934)		40.7 (39.1, 42.2) (2581)	80.9 (74.6, 86.0) (296)	
No	55.7 (54.8, 56.6) (8835)	12.4 (10.9, 14.2) (268)		59.3 (57.8, 60.9) (4318)	19.1 (14.0, 25.4) (61)	
Age at smoking initiation (years, weighted mean ± SD, n)	17.9 ± 0.05 (15,276)	18.8 ± 0.13 (2160)	<0.0001	17.6 ± 0.08 (6880)	19.1 ± 0.30 (348)	<0.0001
Made a quit attempt in the past 12 months			0.0001			0.9439
Yes	36.8 (36.0, 37.7) (5544)	41.6(39.3, 43.8) (887)		35.2 (33.7, 36.7) (2462)	35.4 (30.0, 41.1) (126)	
No	63.2 (62.3, 64.0) (9639)	58.4 (56.2, 60.7) (1292)		64.8 (63.3, 66.3) (4397)	64.6 (58.9, 70.0) (225)	
Non-Daily Smokers						
Number of days smoked out of the past 30 days (mean ± SD, n)	13.6 ± 0.15 (3671)	14.2 ± 0.33 (822)	0.0738	14.2 ± 0.30 (1193)	14.6 ± 0.75 (119)	0.6640
Smoke first cigarette of the day within 30 min of waking			<0.0001			0.0436
Yes	10.4 (9.2, 11.7) (427)	20.7 (17.8, 24.0) (172)		12.7 (10.5, 15.2) (166)	20.1 (11.9, 31.9) (22)	
No	86.5 (85.2, 87.7) (3347)	75.7 (72.2, 78.8) (662)		84.3 (81.5, 86.8) (1055)	74.0 (62.5, 83.0) (91)	
Varies	3.1 (2.5, 3.8) (117)	3.6 (2.5, 5.2) (36)		3.0 (2.0, 4.6) (41)	5.9 (2.8, 12.0) (7)	
Menthol cigarette use			<0.0001			<0.0001
Yes	42.3 (40.5, 44.2) (1607)	84.1 (80.6, 87.1) (744)		41.6 (37.9, 45.4) (479)	88.8 (82.0, 93.3) (104)	
No	57.7 (55.8, 59.5) (2284)	15.9 (12.9, 19.4) (128)		58.4 (54.6, 62.1) (784)	11.2 (6.7, 18.0) (17)	
Age at smoking initiation (years, weighted mean ± SD, n)	19.2 ± 0.11 (3758)	20.7± 0.25 (848)	<0.0001	18.9 ± 0.18 (1224)	21.4 ± 0.68 (118)	0.0005
Former Smokers						
During year before made a quit attempt, smoke first cigarette of the day within 30 min of waking			0.1357			0.1535
Yes	41.2 (39.7, 42.8) (1941)	44.9 (39.9, 49.9) (214)		48.3 (45.5, 51.0) (736)	48.8 (34.9, 63.0) (42)	
No	56.0 (54.3, 57.6) (2538)	51.1 (45.6, 56.6) (241)		49.9 (47.1, 52.7) (740)	45.6 (31.3, 60.7) (32)	
Varies	2.8 (2.2, 3.6) (105)	4.0 (2.5, 6.4) (24)		1.8 (1.2, 2.9) (24)	5.6 (2.3, 13.0) (5)	
Age at smoking initiation (years, mean ± SD, n)	18.2 ± 0.03 (29,922)	19.1 ± 0.11 (2453)	<0.0001	18.3 ± 0.07 (9352)	19.3 ± 0.28 (327)	0.0005
Years since completely stopped smoking (mean ± SD, n)	18.4 ± 0.10 (31,200)	15.5 ± 0.29 (2564)	<0.0001	17.8 ± 0.19 (9723)	15.4 ± 0.84 (345)	0.0050

^1^ Frequencies are unweighted; percentages and statistical tests are weighted. To compare sample characteristics between Black and White individuals living in metropolitan areas and between Black and White individuals living in non-metropolitan areas, Rao-Scott Chi-square tests and F-tests were used for categorical and continuous variables, respectively. ^2^ Tests for differences in current smoking status (i.e., current/former/never).

## Data Availability

Harmonized TUS-CPS data are publicly available on the National Cancer Institute website (https://cancercontrol.cancer.gov/brp/tcrb/tus-cps/questionnaires-data), and other materials can be made available upon request.
